# Multiple Sclerosis in the Mount Etna Region: Possible Role of Volcanogenic Trace Elements

**DOI:** 10.1371/journal.pone.0074259

**Published:** 2013-12-11

**Authors:** Alessandra Nicoletti, Elisa Bruno, Martina Nania, Edoardo Cicero, Silvia Messina, Clara Chisari, Josita Torrisi, Davide Maimone, Roberto Marziolo, Salvatore Lo Fermo, Francesco Patti, Salvatore Giammanco, Mario Zappia

**Affiliations:** 1 Depatment G.F. Ingrassia Section of Neurosciences, University of Catania, Catania, Italy; 2 Neurology Unit, Garibaldi Hospital, Catania, Italy; 3 Neurology Unit, Cannizzaro Hospital, Catania, Italy; 4 Istituto Nazionale di Geofisica e Vulcanologia, Osservatorio Etneo, Catania, Italy; University of Illinois, United States of America

## Abstract

**Background:**

Trace elements have been hypothesised to be involved in the pathogenesis of Multiple Sclerosis and volcanic degassing is the major natural sources of trace elements. Both incidence of Multiple Sclerosis in Catania and volcanic activity of Mount Etna have been significantly increased during the last 30 years. Due to prevailing trade winds direction, volcanic gases from Etna summit craters are mostly blown towards the eastern and southern sectors of the volcano.

**Objective:**

To evaluate the possible association between Multiple Sclerosis and exposure to volcanogenic trace elements.

**Methods:**

We evaluated prevalence and incidence of Multiple Sclerosis in four communities (47,234 inhabitants) located in the eastern flank and in two communities (52,210 inhabitants) located in the western flank of Mount Etna, respectively the most and least exposed area to crater gas emissions.

**Results:**

A higher prevalence was found in the population of the eastern flank compared to the population of the western one (137.6/100,000 *versus* 94.3/100,000; p-value 0.04). We found a borderline significantly higher incidence risk during the incidence study period (1980–2009) in the population of the eastern flank 4.6/100,000 (95% CI 3.1–5.9), compared with the western population 3.2/100,000 (95% CI 2.4–4.2) with a RR of 1.41 (95% CI 0.97–2.05; *p-value* 0.06). Incidence risks have increased over the time in both populations reaching a peak of 6.4/100,000 in the eastern flank and of 4.4/100.000 in the western flank during 2000–2009.

**Conclusion:**

We found a higher prevalence and incidence of Multiple Sclerosis among populations living in the eastern flank of Mount Etna. According to our data a possible role of TE cannot be ruled out as possible co-factor in the MS pathogenesis. However larger epidemiological study are needed to confirm this hypothesis.

## Introduction

The geographic and temporal variations in the frequency of MS have been intensively studied. In the past, a latitudinal distribution of MS, with a north-to-south gradient, constituted one of its epidemiological hallmarks [1]. A recent systematic review of incidence studies of MS has underlined an attenuation of the latitude gradient in MS incidence over the last 25 years, apparently as a result of increased incidence of MS in regions closer to the equator [2]. Several epidemiological studies have been also carried out in Mediterranean countries suggesting a more complex distribution [3]. Concerning the MS distribution in Italy, incidence has steeply increased over the time in several areas [4]–[8], even if in others it has remained stable over the time [9] or increased only for a certain period [10]–[11]. Considering only more recent studies, incidence rates reported in continental Italy are on average about 4.0–5.0 cases per 100,000 per year [4], [5], [8], [9], [10], while higher incidence risks have been generally reported in Sardinia and Sicily [6], [7], [11]. In the city of Catania, incidence has been increasing dramatically during the last 30 years, reaching a peak of 7.0/100,000 during 2000–2004 and, on the basis of the latter estimate, Sicily, along with Sardinia, can be considered the area with the highest risk among the Mediterranean populations [11], [12]–[14].

MS is currently considered a multi-factorial disease, probably due to a complex interaction between susceptible genes and one or more environmental triggers [15]. Among the environmental factors, either excess or deficiency of trace elements (TE) have been hypothesised to be involved in the pathogenesis of various neurologic diseases, including MS [16], [17]. Even if there are several sources of TE in everyday life, volcanoes with their degassing represent the major natural source [18], [19]. Volcanogenic TE are highly toxic in relation both to their concentration and to their redox state and can have serious effects on the environment and on public health in the cities located around an active volcano, as they accumulate in soils, plants and ground waters used for drinking purposes. Mount Etna is the largest active volcano in Europe, emitting a large amount of gases and hence of heavy and alkali metals [19], [20]. Volcanic activity at Mount Etna has increased significantly during the last decades [21], [22], therefore may be reasonable to assume an increasing impact of TE on the populations living at the foot of the volcano. Due to the prevailing westerly to north-westerly trade winds in the Mount Etna region [19], [23], most of the times the volcanic gases from the summit craters are blown to the Eastern and Southern sectors of the volcano. As a matter of fact, in these highly exposed areas, where also rainfall is higher than in the rest of the volcano, the content of TE in plants and in lichens is the highest [23]. Furthermore, the amount of several TE dissolved in Etna ground waters is also significantly higher in the eastern and southern sectors of the volcano. This effect, however, is not directly related with TE deposition from the volcanic plume, but rather it is due to direct input of soluble magmatic gases from depth into the water table through the many deep faults that cross the above sectors of the volcanic edifice and allow flank leakage of magmatic volatiles to the surface [24]–[25]. Therefore, if TE have a role in the pathogenesis of MS, a higher incidence is expected among the populations located in the eastern and southern flanks of the volcano, whereas a lower incidence is expected in the populations located in its western flank, less exposed to crater gas emissions. In order to test this hypothesis we carried out an ecological survey aimed at determining the prevalence and incidence of MS in two populations respectively located in the eastern and western flanks of Mount Etna.

## Materials and Methods

### Ethics statement

The study was approved by local ethical committee (Azienda Universitaria-Ospedaliera Policlinico Vittorio Emanuele, Catania) and patients were enrolled after they signed an informed consent.

### Study area

In order to evaluate the possible link between TE and MS in the Mount Etna area, we assessed the prevalence and incidence of MS among populations most exposed to volcanogenic TE released both through its crater gas plume and through the faults crossing its volcanic edifice, that is those located in its east flank. We also evaluated the prevalence and incidence of MS in populations much less exposed both to crater gas emissions and to TE contents in groundwater, such as those located in the west flank of Mt Etna.

Communities belonging to the two categories above and with a stable population over the time, have been selected according to previous geochemical studies. In particular we have selected an overall population of about 50,000 inhabitants in the most exposed area (east flank of Mount Etna) and a population of similar size for the least exposed area (west flank of Mount Etna).

**Figure 1:**
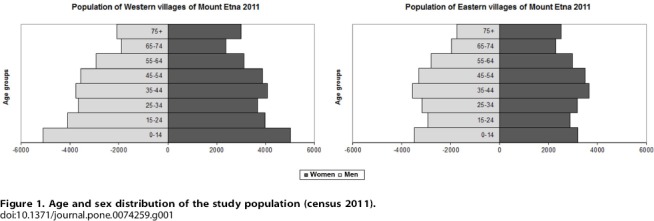
Age and sex distribution of the study population (census 2011).

Finally we selected four communities from the east flank (Giarre, Santa Venerina, Sant'Alfio and Zafferana) and two communities (Adrano and Bronte) from the west flank. Considering the last official census (*census* 2011) [26], the overall population of the communities selected in the eastern flank was 47,229 inhabitants, while that of the communities selected in the western flank was 56,216. According to census data, population in all of the selected communities was quite stable during the last 30 years (*census* 2011) [26]. Age and sex distribution of the study population (census 2011) is shown in figure 1.

### Case ascertainment

The primary sources of MS data investigated were the Neurological hospitals of Catania (Neurological Clinic and MS center of the University of Catania, the Neurological Division and the MS center of the Garibaldi Hospital and the Neurological Division and the MS center of the Cannizzaro Hospital). MS centers in the province of Messina have been also checked. We also requested the list of all the enrolled members of the Italian Multiple Sclerosis Association (AISM).

We further revised the archives until to the end of June 2012 to detect those patients who had their clinical onset of MS during the decade 2000–2009, but who fulfilled the diagnostic criteria only after the study period. Written consent was given by the patients for their information to be stored in the hospital database and used for research. A code has been attributed to each identified cases before the data entry. We verified all sources at least twice. All surviving potential patients with MS identified from these sources, except those attending our neurological department and MS center, were called to undergo neurological examination performed by trained neurologists of our team in order to confirm the diagnosis. Data on deaths were derived from the official “deaths register” of the city of Catania. In order to minimize potential sources of error during data collection, we selected a team of neurology residents who spent at least one month at the MS center and who were previously trained for data abstraction. Information was collected on pre-coded forms created ad hoc and revised by a supervisor. Data were checked for consistency and any error found was corrected.

### Diagnostic criteria

In order to allow comparison with previous surveys [14] and literature data, case definition for the incidence and prevalence estimates was based on Poser's diagnostic criteria [27]. All patients who satisfied these criteria for clinically definite MS (CDMS), laboratory-supported definite MS (LSDMS), clinically probable MS (CPMS) and laboratory-supported probable MS (LSPMS) were considered as prevalent and incident cases.

When available, the onset was established using objective data or the history was obtained interviewing patients and their closest relatives. Prevalence was based on the number of patients who were living in the study area on prevalence day December 31, 2009, and had the clinical onset while living there (onset-adjusted prevalence rate). Crude prevalence rate was age-adjusted to the Italian population (national census 2011) direct method of standardization.

Incidence risk was studied for the three ten-year intervals from 1980 to 2009. Incidence risk was based on the year of the clinical onset (onset-adjusted incidence risk). Confidence intervals for estimates were calculated assuming a Poisson's distribution. For each studied decades incidence risks were also sex and age-adjusted to the Italian population (national census 2011) using the direct method of standardization.

Relative Risk (RR) for each decades were also estimated considering the eastern population as the exposed population and the western population as the unexposed one.

## Results

At the end of the case finding, a total of 118 MS patients fulfilling the Poser's diagnostic criteria were identified in the study areas. In particular 109 (93%) fulfilled the criteria for defined MS (clinically or laboratory supported) while nine (four in the western flank and five in the eastern one) where classified probable MS. Nevertheless all the identified MS patients underwent at least one MRI examination all showing white matter lesions compatible with MS.

### Prevalence

On the prevalence day (January 1, 2009), we detected 53 MS patients (13 men and 40 women) who were resident in the communities located in the western flank of Mount Etna (Adrano and Bronte) and who had experienced the clinical onset of the disease. For this area the onset-adjusted prevalence rate (53 cases with onset before the prevalence day) was 94.3/100,000 (95% CI 68.7–119.3). Age-adjusted prevalence to the Italian population (*census* 2011) was 100,7/100,000. Overall, the prevalence was higher in women (137.3/100,000; 95% CI 99.4–188.9) than in men (48.0/100,000; 95% CI 26.7–84.4).

On the other hand, in the eastern flank communities (Giarre, Santa Venerina, Sant'Alfio and Zafferana), considered the highly exposed area to input of TE, we detected 65 MS patients (20 men and 45 women) who had experienced the clinical onset of the disease on prevalence day. For the eastern area the onset-adjusted prevalence rate was 137.6/100,000 (95% CI 104.5–171.5). Age-adjusted prevalence to the Italian population (*census* 2011) was 137,8/100,000. Again, the overall prevalence was higher in women (230.3/100,000; 95% CI 170.5–305.3), than in men (72.2/100,000; 95% CI 45.3–113.7).

Point-prevalence in the eastern flank population was higher than that in the western flank population (respectively, 137.6/100.000 and 94.3/100,000; p-value 0.04). Both in the eastern and in the western areas age-specific prevalence increased in the first age groups, with a peak in the group aged 35–44 (317.7/100,000 and 216.7/100,000 respectively) and it showed a steep decline in the other groups.

In the western flank population, the mean age at onset was 29.2±9.5 years, with a mean disease duration of 12.6±8.1 years, whereas in the eastern flank population the mean age at onset was 30.2±10.6 years with a mean disease duration of 13.5±9.3 years.

None of the 118 identified MS patients reported some kinship up to the second-degree relatives (grandparents).

### Incidence

During the incidence study period (1980–2009) 51 MS patients (13 men and 38 women) living in the western area of the Mount Etna had the clinical onset of the disease. During the incidence study period the average population in the western area was 52,561 (25,724 men and 26,837 women). The average annual onset-adjusted incidence was 3.2/100,000 (95% CI 2.4–4.2), 2.8/100,000 sex and age-adjusted to the Italian population. Incidence was 4.7/100,000 (95%CI 3.3–6.4) for women and 1.7/100,000 (95% CI 0.9–2.9) for men. In the western flank population the incidence risk increased from 1.0/100,000 (95% CI 0.3–5.6) during 1980–1989 to 4.2/100,000 (95% CI 2.6–6.5) during 1990–1999, then remaining quite stable during the following decade 2000–2009 (4.4/100,000; 95%CI 2.8–6.5).

Concerning the eastern flank population, from January 1980 to December 2009, 59 MS patients (19 men and 41 women) had the clinical onset of the disease. During the incidence study period, the average population in the eastern flank area was 43,797 (23,511 men and 20,284 women). The average annual onset-adjusted incidence was 4.6/100,000 (95% CI 3.1–5.9), 3.9/100,000 sex and age-adjusted to the Italian population. Incidence was 6.7/100,000 (95%CI 4.8–9.1) for women and 2.7/100,000 (95% CI 1.6–4.2) for men. In the population living on the eastern flank of Mount Etna the incidence risk increased from 1.7/100,000 (95%CI 0.7–3.5) during 1980–1989 to 5.5/100,000 (95%CI 3.5–8.1) during 1990–1999, reaching a peak of 6.4/100,000 (95%CI 4.3–9.1) during the decade 2000–2009.

**Figure 2:**
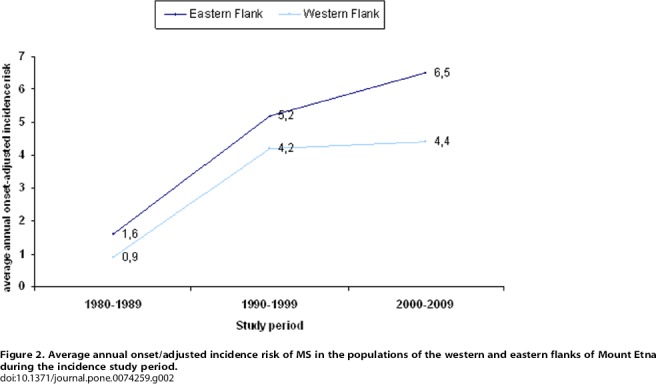
Average annual onset/adjusted incidence risk of MS in the populations of the western and eastern flanks of Mount Etna during the incidence study period.

Average annual incidence risk for 10-year intervals from 1980 to 2009 both for the western flank and for the eastern flank populations are shown in table 1 and figure 2. We found a borderline significantly higher incidence risk during the incidence study period (1980–2009) in the population located in the eastern flank of Mount Etna, compared with the population located in the western flank (RR 1.41; 95% CI 0.97–2.05; *p-value* 0.06). In particular, the risk ratio (RR) for the first decade (1980–1989) was 1.69 (95% CI: 0.54–5.32; *p-value* 0.3), it was 1.32 (95% CI: 0.74–2.35; *p-value* 0.3) for the second decade (1990–1999), while it was 1.45 (95% CI: 0.84–2.48; *p-value* 0.1) for the last decade (2000–2009).

**Table 1:** Average onset adjusted incidence of MS in the populations of the western and eastern flanks of Mt. Etna during the incidence study period.

	**Incidence of MS in the western flank**									
**Years**	**Men**	**Cases**	**Cases/100000**	**Women**	**Cases**	**Cases/100000**	**Ave Total**	**Cases**	**Cases/100000**	Cases/100000**
80–89	25115	/	/	25454	5	2.0	50569	5	1.0	0.8
90–99	25364	6	2.4	26851	16	6.0	52215	22	4.2	3.7
00–09	26333	7	2.7	28221	17	6.0	54554	24	4.4	3.9
**Total**	25724	13	1.7	26837	38	4.7	52561*	51	3.2	2.8

	**Incidence of MS in the eastern flank**									
**Years**	**Men**	**Cases**	**Cases/100000**	**Women**	**Cases**	**Cases/100000**	**Total**	**Cases**	**Cases/100000**	
80–89	20526	3	1.5	21501	4	1.9	42027	7	1.7	1.1
90–99	23011	11	4.8	20201	13	6.4	43212	24	5.5	5.1
00–09	26497	5	1.9	19068	24	12.6	45568	29	6.4	5.6
**Total**	23511	19	2.7	20284	41	6.7	43797*	60	4.6	3.9

Legend:

* Average population 1981–2011;

** sex and age-adjusted to the Italian population *(Census 2011)*.

Overall the mean length of time between the date of clinical onset and the date of diagnosis significantly decreased from 6.9±6.2 years during 1980–1989 to 3.5±3.5 years during 1990–1999 and 2.6±2.4 years during 2000–2009. The female-to-male ratio for the entire incidence study period was 2.77 in the western flank (95% CI 1.47–5.19; *p-value* 0.0009) and 2.50 in the eastern one (95% CI 1.45–4.30; *p-value* 0.0006). Age at onset steeply increased from 22.2±8.3 years during 1980–1989 to 29.7±9.1 years during 1990–1999 and 31.8±10.7 years during the last decade without significant difference between the two flanks. Out of the 118 MS patients, 9 (1.7%) had had the onset before the age of 10 (5 in the western flank and 4 in the eastern one).

## Discussion

In the city of Catania, incidence has been increasing dramatically during the last 30 years, from 1.3/100,000 during the period 1975–1979 to 7.0/100,000 during the period 2000–2004 [14]. This figure is close to the incidence reported in Sardinia [6], [11], where probably a different genetic background contributes to the elevated MS risk, but it is higher than those recently reported for continental Italy [4], [5], [8], [9], [10]. Considering that the population of Catania is ethnically stable, the increasing incidence over the time during the last 30 years strongly suggests a change in the exposure to one or more exogenous causative factors for MS over time.

MS is a complex disease that is due to interactions between genes and environmental factors that lead to tissue injury by autoimmune mechanisms. The environment influences MS risk at a population level. Important environmental factors include sunlight exposure, vitamin D status, smoking and infections. Of the infectious agents linked to MS, epidemiological evidence currently supports a role for EBV infection in the pathogenesis [28].

Literature provides evidence of a possible role of environmental and occupational factors such as exposure to organic solvents, to Zn used in manufacturing plant, to Ba contaminant in the ecosystems or presence of Co, Mn, Mo and Zn in arable soils [29]–[32]. Furthermore, several trace or major elements have been investigated and increased levels of Cd, Cu, Fe and Pb, together with a decrement of Mg and Zn, have been reported among MS patients [33]. Additional studies have shown variations in Cu, Mg and Zn concentrations in serum and cerebrospinal fluids from patients affected by MS [34], [35]. Studies performed so far highlighted the possible role of TE into the MS pathogenesis and disease development without allowing definitive conclusions about the exact relationship between metals and the pathology.

Alterations in metal homeostasis might result in an increased level of free radicals [36]. A large body of evidence implicates crucial roles of oxidative stress in the pathogenesis of several neurodegenerative disorders including MS [37]. The progressive myelin destruction may be accelerated by oxidative stress and metals in fact may exert or aggravate toxic effects on myelin. Toxic effects are of course more likely with long-term overexposure [38]. On the other hand cytokines production can be influenced by various chemicals and among metal Ni, Cr, Cd, Co and Hg are especially known to influence immunocompetent cells [39].

For instance exposure to Ni compounds can have adverse effects on human health [40]. The possible role of Ni in inducing oxidative stress has been well demonstrated in animal model and it has been also demonstrated that endothelial surface molecules (such as ICAM-1 and VCAM-1) are upregulated by NiCl_2_ exposure in cultured human cells [40].

Natural sources of heavy metals can play an important role and active volcanoes release considerable amounts of metals and semimetals as the result of their very high volatility in high temperature magmatic vapours [19]. Mt. Etna is the largest active volcano in Europe, emitting about 16% of the global volcanic heavy metals (including nickel, cadmium, lead) and 19% alkali metals (including sodium, potassium, lithium) during eruptions and less than 5% during quiet periods [41]. It should be noted that, for example, Mount Etna emits about 100 tons/year of Ni [19]. The general population is mainly exposed to Ni through food intake and inhalation from the air and a possible additive source of Ni exposure in the local population may come from the emission of Ni in the soil or in the volcanic plume or ashes [19]. Furthermore, volcanic activity at Mt. Etna has increased significantly during the last decades [21], [22], both in terms of frequency of eruptions and in terms of lava emission rate, thus releasing a huge amount of TE in the environment.

The almost concurrent increase both of MS cases and of frequency of eruptions gives rise to a first assumption of a possible correlation between the two phenomena. Due to the higher exposure of the populations living on the eastern flank of the volcano compared to those living on its western flank, it is expected to find a higher occurrence of MS in the former than in the latter. In agreement with our hypothesis, a higher frequency of MS, both in terms of prevalence and incidence, has been recorded in the populations located on the eastern flank of Mount Etna. Actually, the incidence risk found in the eastern flank populations is close to that reported in the city of Catania (7.0/100,000), that is also located in the eastern-southern area. Conversely, the incidence risk found in the populations located on the western flank (4.4/100,000 during the last decade) is close to those recently reported for continental Italy [4], [5], .

Nevertheless it should be noted that incidence mainly increased in both flanks from the first to the second decade (from 1.0/100,000 to 4.2/100,000 in western flank and from 1.7/100,000 to 5.5/100,000 in eastern flank). This change in temporal trend could be considered the effect of improvement in diagnostic techniques and medical facilities as reflected by the shortened lagtime during the same period (from 6.9±6.2 to 2.6±2.4 years). In our population the mean age at onset increased steadily over the incidence study-periods from a mean of 22.2±8.3 to 31.8±10.7. A similar trend has been already observed by our group in the city of Catania as well as in Sardinia [11]. In agreement with literature data female-to-male ratio over the entire study period was 2.7 without significant differences in both flanks [2]. However it should be noted that incidence estimates for each single stratum (by decade and sex) could be affected by random variation related to the small number of events and for this reason further stratified analysis could produce not reliable estimates.

Ecological studies look for associations at the group level rather than at the individual level and, therefore, we do not have information on the outcome status of exposed versus non-exposed individuals within the same group. However, several limits related to the ecological design should be taken into consideration in the interpretation of our findings. Ecological studies are particularly susceptible to information bias, therefore detecting and controlling confounding factors can be extremely difficult. Several factors are in fact more strongly associated with each other at the group level than they are at the individual level. If the exposure we are studying is highly correlated with a potential confounder, it can be difficult or even impossible to separate out the effect of exposure alone. Groups usually differ in many factors and it can be very difficult to judge which factor is responsible for differences in outcomes between groups. For this reason we have compared groups selected from the same province (i.e., that of Catania) that are quite similar (in terms of genetic background, UV exposition, lifestyle, diet, etc.), except for the exposure of interest (in this case, exposure to TE).The main weakness of an ecological study is the so called “ecological fallacy”. In this respect, ecological studies may allow to make ecological inferences about effects at the group-level, but they do not enable us to make inferences about individual risks. On the other hand, ecological studies can often be carried out in a relatively quick and cheap way, using routine or secondary data and they can be used as a first step in the investigation of a possible exposure-outcome relationship.

Nevertheless, despite the evidence supporting the possible association between TE and MS, the reported excess MS cluster in the small town of Linguaglossa on the lower north flank of the volcano [42], [43] and the proven presence of elevated levels of certain TE in the Mount Etna region, this study represents the first attempt to investigate the possible direct link between exposure to volcanogenic TE and occurrence of MS in this region.

The aetiology of MS is still unknown. Genetic, environmental and immunological factors (smoking, viruses, vitamin D etc.), have been implicated in the aetiology of this complex, multifactorial and heterogeneous disease and in this *scenario* it is also possible that the chronic exposure to high levels of TE can play a role. We are aware that other hidden causes of human exposure to TE are present in everyday life, therefore the study of a “natural model” of volcanic heavy metals emission in the environment could represent an important source of information, complementary to the broader and widespread model represented by industry-related TE.

In conclusion, according to our data a possible role of TE cannot be ruled out as possible co-factor in the MS pathogenesis. However larger epidemiological study are needed to confirm our results and population-based case control studies focused on measuring the TE levels in biological samples should be performed in order to strengthen this hypothesis.
