# Correction: Multiple Sclerosis in the Mount Etna Region: Possible Role of Volcanogenic Trace Elements

**DOI:** 10.1371/journal.pone.0100942

**Published:** 2014-06-16

**Authors:** The PLOS ONE Staff

The authors would like to include an Acknowledgements section in the article and thank Drs Claudia Cascio, Parvez Haris and Richard Jenkins from the De Montfort University, Leicester (UK), for their contribution in the generation of the original hypothesis on the possible link between the volcanogenic trace element and multiple sclerosis. The present epidemiological study has been developed from a previous collaborative research project (1-3).

1. Cascio C, Rodriguez-Lado L, Polya DA, Zappia M, Patti F, Nicoletti A, Jenkins RO, Haris PI. Geogenic trace elements in groundwaters in the Mt Etna region: Geostastistical, proteomic and epidemiological approaches to assessing human exposure and health risks GEOCHIMICA ET COSMOCHIMICA ACTA Volume: 73 Issue: 13 Pages: A197-A197 Published: JUN 2009.

2. Cascio C. (2011) PhD thesis: A multidisciplinary study of human exposure to arsenic and other trace elements (https://www.dora.dmu.ac.uk/handle/2086/5411).

3. Nicoletti A, Cascio C, Bruno E, Jenkins R, Lo Fermo S, Messina S, Morton J, Patti F, Quattrocchi G, Haris P, Zappia M (2012). Volcanogenic trace elements and MS: a case control study on the Linguaglossa cluster in the Mount Etna region. NEUROLOGICAL SCIENCES, In: Neurological sciences. vol. 33 (supp), p. S152-S153, ISSN: 1590-3478).

## References

[1] Nicoletti A, Bruno E, Nania M, Cicero E, Messina S, et al (2013) Multiple Sclerosis in the Mount Etna Region: Possible Role of Volcanogenic Trace Elements. PLoS ONE 8(12): e74259 doi:10.1371/journal.pone.0074259 24348986 10.1371/journal.pone.0074259 PMC3859652

